# Evaluation of cloned cells, animal model, and ATRA sensitivity of human testicular yolk sac tumor

**DOI:** 10.1186/1479-5876-10-46

**Published:** 2012-03-13

**Authors:** Junfeng Zhao, Congde Chen, Haochuan Zhang, Jinhui Shen, Hua Zhang, Xiaokun Lin, Le Qin, Xiaozhou Bao, Jie Lin, Wenqiang Lu, Xiangdong Wang, Xiaoming Chen

**Affiliations:** 1 Department of Pediatric Surgery, the Second Hospital, Wenzhou Medical College, Wenzhou, China; 2 Institute of Translational Medicine, the First Hospital, Wenzhou Medical College, Wenzhou, China; 3 The First Hospital, Wenzhou Medical College, Wenzhou, China

**Keywords:** Testicular yolk sac tumor, Human, Clone, Model, ATRA, Cisplatin

## Abstract

The testicular yolk sac tumor (TYST) is the most common neoplasm originated from germ cells differentiated abnormally, a major part of pediatric malignant testicular tumors. The present study aimed at developing and validating the in vitro and vivo models of TYST and evaluating the sensitivity of TYST to treatments, by cloning human TYST cells and investigating the histology, ultra-structure, growth kinetics and expression of specific proteins of cloned cells. We found biological characteristics of cloned TYST cells were similar to the yolk sac tumor and differentiated from the columnar to glandular-like or goblet cells-like cells. Chromosomes for tumor identification in each passage met nature of the primary tumor. TYST cells were more sensitive to all-trans-retinoic acid which had significantly inhibitory effects on cell proliferation. Cisplatin induced apoptosis of TYST cells through the activation of p53 expression and down-regulation of Bcl- expression. Thus, we believe that cloned TYST cells and the animal model developed here are useful to understand the molecular mechanism of TYST cells and develop potential therapies for human TYST.

## Introduction

The testicular yolk sac tumor (TYST) is the most common neoplasm originated from germ cells differentiated abnormally [1], while germ cell tumors in the testis account for approximately 60-75% of pediatric malignant testicular tumors. The yolk sac tumor as endodermal sinus tumor is a common malignant tumor accounting for 1-2% of cancers in men and one of the most common types of cancer in young men between 15-35 ages. Of them, the TYST mainly occurs in neonates and infants, different from adolescences or adults who composed of multiple germ cells and having own biological characters [2].

The TYST is still a highly malignant neoplasm with poor prognosis, increased resistance to chemotherapy, recurrence after initial chemotherapy or surgery, and the side effects of chemotherapeutics, even though the survival rate of patients with TYST was improved after surgical resection or platinum-based combination chemotherapy, e.g. cisplatin, etoposide and bleomycin [3]. The regulation of cell differentiation from immature malignant tumor cells to mature was suggested as a potential therapy for tumors [4]. Conventional radiotherapy and/or chemotherapy were found to suppress the bone marrow and immune function through influencing cell phenotypes [5]. The cell apoptosis is closely related with the tumorigeness, tumor development and insensitivity of chemotherapy/radiation therapy [6].

There are limited studies on human TYSTs, although YST has been studies in cells from male murine embryonal carcinoma in vitro [7] and ovarian YST cell lines [8]. The present studies aimed at establishing the animal model of TYST and the human TYST cell line and evaluating the characteristics of the disease and bio-function of human TYST cells. The present study evaluated the role of ATRA as an inducer of differentiation in a variety of tumor cells in the growth TYST cell lines in vitro and explored the molecular mechanism of TYST cell proliferation. Effects of cisplatin on TYST cell apoptosis and the expression of P53 and Bcl-2 genes were furthermore investigated.

## Materials and methods

### TYST and sampling

TYST tissues were sampled children with TYST, aging about 2-3 years-old, during the testicular surgery, without any radiotherapy or chemotherapy. The study protocol and informed consent of the sampling for scientific research were approved by The Ethical Committee of Clinical Research of Second Affiliated Hospital of Wenzhou Medical College. Informed written consents were approved from guardians on the behalf of the children participants involved in the study. Tumors with diameters about 50-70 mm and without the encapsulation were severely adhered with the surrounding tissues. Plasma levels of alpha-fetoprotein were above 1200 ng/ml, corresponded with the normal reference value of 0-7 ng/ml. TYST samples were positive in immunohistochemical staining against cytokeratins and alpha-fetoprotein.

### A xenograft tumor model

Male BALB/C mice with the autosomal recessive nude gene in homozygous (nu/nu), aging four weeks, were purchased from Shanghai Experimental Animal Center of Chinese Academy of Sciences. Mice were housed and maintained in individual ventilation cabinets under specific pathogen-free conditions with constant temperature at 24°C. Animal studies were approved by the local ethical committee for animal care according to international guidelines and regulations for use and care of animals. Human tumor specimen from the surgery was washed immediately and sliced into near 1 mm^3 ^masses under sterile conditions. Each piece of tumor mass was implanted hypodermically into the unilateral inguinal region in mice. Tumors growth was observed periodically and mice were terminated until the tumor grew to 2-3 cm in diameter. Tumors were exteriorized and implanted in new mice as described, and then perpetuated in mice by consecutive passages from the primary tumor. This study was carried out in strict accordance with the recommendations in the Guide for the Care and Use of Laboratory Animals of the National Institutes of Health. The protocol was approved by the Ethical Committee on Animal Experiments of the Wenzhou Medical College. All surgery was performed under anesthesia of sodium pentobarbital to minimize suffering.

### Measure of tumor growth

The latent period and growth rate of tumor were recorded daily. Volumes and exponential function of tumors were measured weekly from the day when tumors reached 3 mm in diameter. Volume of tumors (Vt) was calculated in the formula: Vt = π(b2 × a)/6 (b and a represent the minimal and maximal diameter in millimeters, respectively) and exponential function (K) was calculated as growth rate: K = (InVt-InV0), and then doubling time: Td = In2/K.

### TYST morphologies

Tumors were obtained from surgical resection, fixed in 10% formalin, embedded with paraffin, and sectioned in a thickness of 4 um. Slices were then de-paraffinized, rehydrated, and finally stained with hematoxylin and eosin. The tumor morphology was analyzed and observed under light microscope. For ultrastructural analysis, tumors were cut into small pieces about 1 mm^3^, immersed and fixed in a solution of 4% glutaraldehyde (pH 7.3, 4°C). Fixed samples were washed in phosphate buffer twice and then post-fixed in 1% osmium tetroxide (Polysciences Inc., New Orleans, LA, USA) for 1 hr. Samples were then rinsed extensively in distilled water, dehydrated in a graded series of ethanol, embedded in Eponate 812 resin (Ted Pella Inc., Redding, USA), and dried by heat with a graded temperature. Sections of 50 nm were then cut with a Leica Ultracut UCT ultramicrotome (Leica Microsystems Inc, LKB-II, Germany), stained with 3% solution of uranyl acetate and lead citrate, and mounted on mesh grids. Digital pictures (2048 × 2048 pixels, 4 MB, and uncompressed grayscale Tiff files) were obtained using a high resolution digital camera MegaViewIII (SIS^®^) connected to the TEM, and observed at electron microscope with an acceleration voltage of 80 kV, in JEOL JEM-1230 (Japan).

### Immunohistochemistry

TYST Tumors were fixed in formalin for approximately 10 hrs, embedded in paraffin overnight in a routine fashion, and cut into sections at the thickness of 4 μm. Sections were subsequently boiled for 10 minutes in 10 mM citrate (pH 6.0), after being dewaxed and incubated in methanol containing 0.3% H_2_O_2 _for 15 minutes. Sections were digested with 0.25% pepsin (Sigma) dissolved in 0.1 M HCl for 15 minutes at 37°C, blocked for 30 minutes in PBS containing 5% normal mouse serum (Jackson Immunoresearch, Newmarket, UK), and then incubated with antibodies again alpha-fetoprotein (AFP, goat anti-mouse alpha-fetoprotein polyclonal antibody; R&D Systems, Minneapolis, USA), placental alkaline phosphatase (PLAP, anti-placental alkaline phosphatase antibody, Abcam, Cambridge, UK), or cytokeratins (CK, mouse anti-mouse cytokeratin 10 monoclonal antibody, unconjugated, clone Spm262; Thermo Fisher Scientific, Rockford, IL, USA) for 2 hours, while HRP-conjugated secondary antibodies for 30 minutes, both at room temperature. TYST sections were stained using 3,3'-diaminobenzidine and alternatively counterstained with haematoxylin. The concentrations of primary antibodies were used at 1:100. TYST sections were observed and photographed under an inverted Olympus phase contrast microscope equipped with a digital camera (Olympus dp71, Japan). For the analysis of positive cellular numbers (PCN) and the optical density (OD), total 20 hotspot fields were captured for each section in viable zones at × 400 using a camera and analyzed by image-pro plus 6.0 software. Semi-quantitative analyses of immunohistochemical staining were performed as the formula of expression values (EP): = PCN × OD.

### Chromosomes analysis

Fresh TYST samples were obtained, washed with PBS twice, sliced into small particles, and then put into culture medium (RPMI 1640, L-glutamine 2 mM, penicillin 100 units/ml, streptomycin 100 mg/ml, colcemid 0.1 pg/ml) without serum. These particles were put into colchicine and incubated in the water bath at 37°C for 1 h. Tumor cells were centrifuged, fixed, and detected in G-banding. Chromosomes were counterstained and reverse banded by mounting the slides in Citifluor antifade AFl (Citifluor Ltd) containing 2.5 μg/ml of DAPI and 0.5 μg/ml of propidium iodide, as described in previous studies [9]. Hybridised slides were assessed using a Nikon Optiphot fluorescence microscope with a × 100. Images were recorded as grey levels at two detectors (detector 1, 500 to 560 nm; detector 2 < 600 nm) of an MRC 600 confocal scanning head (Biorad) and displayed in a pseudo-color (FITC fluorescence in green, PI fluorescence in red). The band location of FITC signal was determined by toggling the FITC signal to allow the banding pattern beneath the signal.

### Primary culture of TYST cells and establishment of cell line

TYST tissues were harvested, immediately washed by D-Hank's solution (NaCl 8.0 g, KCl 0.2 g, Na_2_HPO_4_·H_2_O 1.56 g, KH_2_PO_4 _0. 2 g in 1 L distilled water) in order to clean up blood and mucus in surface, and then sliced into 1 mm^3 ^pieces. Sliced tissues incubated in Dulbecco's modified Eagle medium (GibcoBRL, Grand Island, NY, USA) solution containing 20% fetal calf serum at 37°C. The first generation of cells was passed when cells grew to cover the 80% bottom of the culture bottle in 14 days, and the second passage in 8 days. From the third generation of cells, the average passage time was 3 to 4 days until 25 generation and cells grew stably and kept the primary characteristics. Cells from the 8th generation were used in the study and the survival rate of recovery cells from freezing reached about 80% and their morphologies were observed under inverted microscope, light microscope and electron microscope, respectively.

### Bio-function measurement

Cell growth curve was drawn by counting and recording cell density of three holes, of which each was operated with 3 times interval 24 hours during days 1-8. Cells were diluted at the density of 2 × 10^4 ^per ml and inoculated into 24-well plate. The single cell suspension was made and cell doubling time was monitored. Cytogenetic analysis was performed in TYST cells in the logarithmic growth phase, after cells were treated with trypsin, fixed with methanol-acetic acid solution, and stained with trypsin-Giemsa. About 100 cells in metaphase and the number of chromosomes and its modes were accounted [10], after then pictures were captured by Applied Imaging Software with G-banding analysis. The expression of AFP and beta-subunit human chorionic gonadotrophin (β-hCG) was measured by immunocytochemical staining according to the manufacture recommendation.

### DNA ploidy analysis

Cellular DNA ploidy and cycle were detected by flow cytometry, as described previously [11]. Briefly, DNA ploidy was analyzed after the cell sorting, after the flow cytometry was calibrated with fluorescent DNA-Check Beads (Coulter) to obtain a percentage half peak coefficient of variation (%HPCV). Histograms were recorded for a minimum of 10,000 nuclei, according to the guideline criteria drawn up in the DNA Cytometry Consensus Conference [12]. The exclusive criteria were the coefficient of variation greater than 8% or background debris constituting more than 20%. DNA diploidy was defined by the presence of a single G0/G1 peak to a histogram. A tumor was considered as aneupoid if a histogram had two separate G0/G1 peaks. The DNA index was calculated from the ratio of the model channel numbers of aneuploid peaks to the modal channel numbers of the diploid peak. Intratumoral DNA heterogeneity was defined by the presence of both DNA diploidy and aneuploidy with a tumor, or by the presence of multiple stem lines in aneuploid patterns. DNA histograms were assessed using MultiCycle software version 2.5 (Coulter) to determine the SPF. A polynomial modeling system was used for cell cycle analysis.

### Cloning procedure

Cells at the 26th passage were cultured in soft agar and the rate of cell clones formation was tested. Briefly, cells were cultured in the medium containing high glucose Dulbecco's modified Eagle medium supplemented with 10% fetal bovine serum (Hyclone, Utah, USA). Two days after cell passage, the medium was transferred into a sterile tube (Corning Incorporated, Corning, NY, USA), centrifuged at 2000 rpm for 10 min. Cells grew to approximately 80% confluence, and the culture flask was placed in 4 refrigerator for 4 h to synchronize the cells, followed by incubation overnight. The single cell suspension was prepared after trypsinization (2.5 g/L trypsin, Difco, prepared in Ca^2+ ^and Mg^2+ ^free Hanks solution d-Hanks). The cell viability was confirmed by trypan blue exclusion, and cloning procedure was performed using the limited dilution method [6]. The cloned cells were preserved in culture medium containing 100 g/L dimethyl sulfoxide (DMSO, Sigma Chemical Co, St Louis, MO, USA), and stored in liquid nitrogen till used for in vivo screening.

### Role of retinoic acid

Cloned TYST- cells were randomly divided into five groups (n = 10/group) treated with vehicle (group 1) or ATRA at concentrations of 0.01, 0.1, 1.0 or 5.0 uM (groups 2-5). ATRA was solved in ethanol at the start and diluted with PBS at the final concentration of 0.1% as vehicle. Cells were seeded in 6-well culture plates at 2 × 10^4 ^for 24 hours and the original medium was replaced by the different concentrations of ATRA solution. Cells growth was monitored under inverted microscope daily for 7 days. Inhibitory effects of ATRA on TYST cell proliferation were detected by MTT assay at 24, 48 and 72 hours, respectively. The absorbency was measured with the colorimetric microplate reader at the wavelength of 570 nm. The inhibitory rate was calculated as the following formula: (%) = (1- experimental control group OD/blank control group OD) × 100%. The expression of retinoic acid receptor β (RAR-β) in cells was measured by RT-PCR, after total RNAs were extracted in each experimental group, according to manufacture's protocol. The synthesis of cDNAs was followed by reverse transcriptase kit protocol. Human RAR--β primer sequences include: forward primer 5'-GAGGACTGGGATGCCGAGAA-3' and reverse primer 5'-TTGACCCAAACCGAATGGC-3'; and human β-actin: forward primer 5'-TCGACAGCTCCGGCAT-3' and reverse primer 5'-AAGGTGTGGTGCCAGATTTTC-3'. Twenty-two cycles of amplification with denaturation at 94°C, annealing at 60°C and extension at 72°C for 60s each, were carried out after the initial pre-denaturation at 95°C for 5 min. The relative expression of targeted mRNA = the product of targeted mRNA with the volume of gary/the product of β-actin with the volume of gray.

### Role of cisplatin

Cloned TYS cells at the 6th passage were treated with cisplatin at the concentration of 2 μg/ml for 12, 24, 48 and 72 hours, respectively. Acridine Orange/Ethidium Bromide (AO/EB) staining fluid at the concentration of 100 mg/L was added into 96-wells at the volume of 20 μl. Cells were incubated at dark for 20 min and observed under fluorescence microscope. Cell apoptosis was analyzed with terminal deoxynucleotidyl transferasemediated biotin-dUTP nick end labeling (TUNEL) staining (Merck). Briefly, specimens were incubated with proteinase K at 20 μg/ml for 15 min at room temperature and washed with PBS for 5 min thrice. Sections were blocked with 3% bovine serum albumin and 20% fetal calf serum solution for 30 min, and incubated with TUNEL reaction mixture of TdT and dUTP (1:9) at 37°C for 1 h. Finally, samples were stained with DAB under control of microscope, and counterstained with hematoxylin for 1 min. Total five fields were captured for each section under microscope. The apoptotic index (AI) = the positive cell numbers/total cell numbers. Expression of AFP, P53 and Bcl-2 proteins was detected with immunohistochemical staining. Mouse monoclonal antibodies of AFP, P53 and Bcl-2 were diluted with 1:100. Positive cellular numbers, optical density, and total ten fields captured for each section in viable tissue zones using a camera (×400, Nikon Digital Sight DS-L2, Japan), were analyzed by Image-pro plus6.0 software, respectively. The semi-quantitative = optical density × cellular numbers.

### Statistical analysis

Data are presented as means ± standard deviation. All experiments were performed twice and 3-5 animals in a group were randomly allocated into each study. After statistical analysis demonstrated no significance between studies, the results from the same group (n = 6-10) were pooled. The difference between groups and time points was calculated using the unpaired, two-tailed Student t-test after the analysis of one-way ANOVA. Inhibitory effects (%) were calculated as the formula: inhibitory rate (%) = [(values of MMT density in different time points treated with different concentrations of inhibitors - values of MMT density in different time points treated with vehicle) - values of MMT density in different time points treated with vehicle] × 100. A p-value of less than 0.05 was considered significant.

## Results

**Figure 1:**
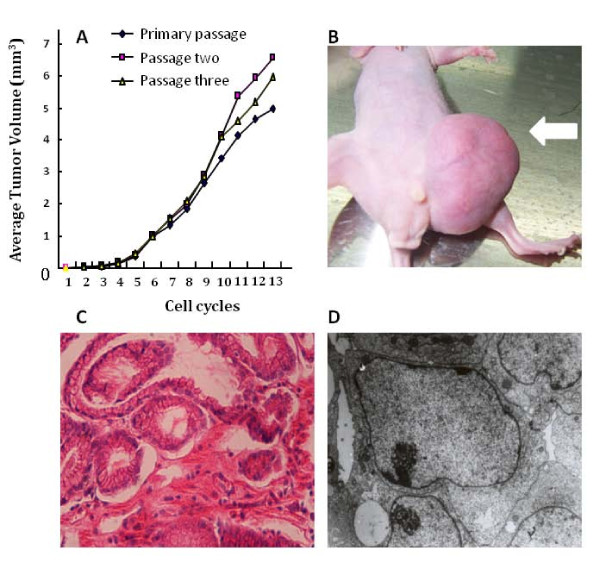
**Average volumes of tumor driven from the primary generation, the first generation and the second generation of testicular yolk sac tumor (TYST) cells were compared with the number of cell cycles (A)**. The tumor was formed from the generation four of TYST cells and on after the subcutaneous transplantation (B). Histogram of tumor formed from cloned TYST cells stained with HE (C, × 400). Ultrastructure of cloned TYST cells with basement membrane materials, oval and enlargement nuclei, and glandular-like structures between cells (**D**, × 5000).

Average volumes of tumor driven from the primary generation, the first generation and the second generation of TYST cells were increased, as shown in Figure 1A. Tumor cell volumes of three generations started to show different from the 10 cycles. Consecutive transplantation of total 7 generations into the subcutaneous inguinal region of nude mice was performed during 15 months. The rate of tumor formations was 20, 40 and 65% in the generations one, two and three, respectively, and reached 100% from the generation four and on after the subcutaneous transplantation (Figure 1B). The average period of tumor formation was 32 days and the maximal time of tumor growth was 192 days in mice. Tumor could grow to about 32 × 28 mm^3 ^in volume, of which some had the ulceration and necrosis on the surface of TYST and lead to cachexia in nude mice. All tumors represented the substantial neoplasm without cystic or mixed ones.

Tumors and internal organs, like liver and lungs, were harvested and stained immediately after tumor-bearing mice were sacrificed (Figure 1C). TYST cells had enlarged volume, obvious heteromorphism, large nucleolus with stained deeply and disordered, abundant cytoplasm with pale staining. The nuclei appeared round or oval, arranged like the glomerular structure and loose mesh-like structure. Some of TYST cells with the papillary-like structure surrounded small blood vessels where inner walls were also covered by malignant cuboidal or columnar cells with the tube-like structure named Schiler-Duval bodies as the most distinctive histological feature. No metastasis was noticed in the liver and lung of tumor-bearing mice.

TYST cells were mainly located between poorly differentiated and undifferentiated state, of which the most exhibited the embryonic or primordial ones, with many glandular-like structures, large nucleus in oval shape, nucleoli prominent, or reduction of intracellular organelles. TYST cells with microvillus structure were partially differentiated into the epithelium-like cells characterized by vacuolar goblet with mucus-like substance. Of TYST cells, some differentiated well were rich in intracellular organelles and nuclei stained deeply, contained desmosomes, or had junction complexes. There were abundant with rod-like crystals, mucus-like substances and ribosomes in differentiated intestinal epithelial cells, which indicated the active proliferation and strong protein synthesis of cells. AFP could be detected outside of cells, which represented the composure of electron dense material as basement membrane material (Figure 1D).

**Figure 2:**
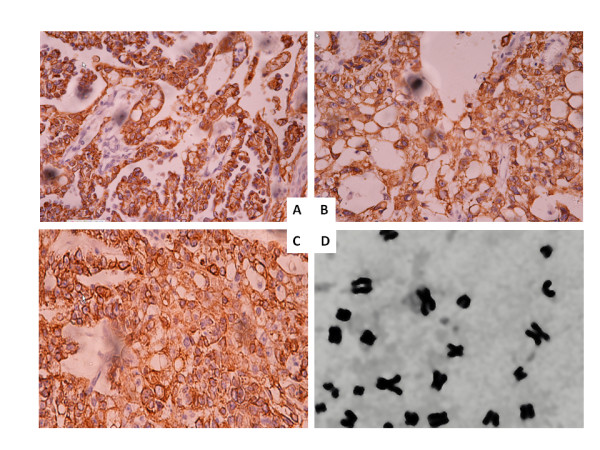
**Histogram of the expressions of alpha- fetoprotein (A), placental alkaline phosphatase (B) and cytokeratins (C) in the tissue formed from cloned testicular yolk sac tumor cells and stained by Immunohistochemistry**. Chromosomes with the diploid structure in cloned testicular yolk sac tumor cells (**D**). The origin magnification was × 400.

Figure 2 demonstrated the expressions of AFP (A), PLAP (B) and CK (C) in TYST tissue by immunohistochemical staining. Values of average optical density of AFP, PLAP and CK staining at 0.13 ± 0.08, 0.10 ± 0.06 and 0.13 ± 0.07 (n = 90-100 sections per staining), respectively, were significantly higher than those in the group without primary antibodies (0.04 ± 0.01). The number of chromosomes with 34-48 and the modal number 46 were consistent with the diploid structure in tumors, different from those of mouse or human (Figure 2D). The breakage or loss was noted in some of chromosomes.

**Figure 3:**
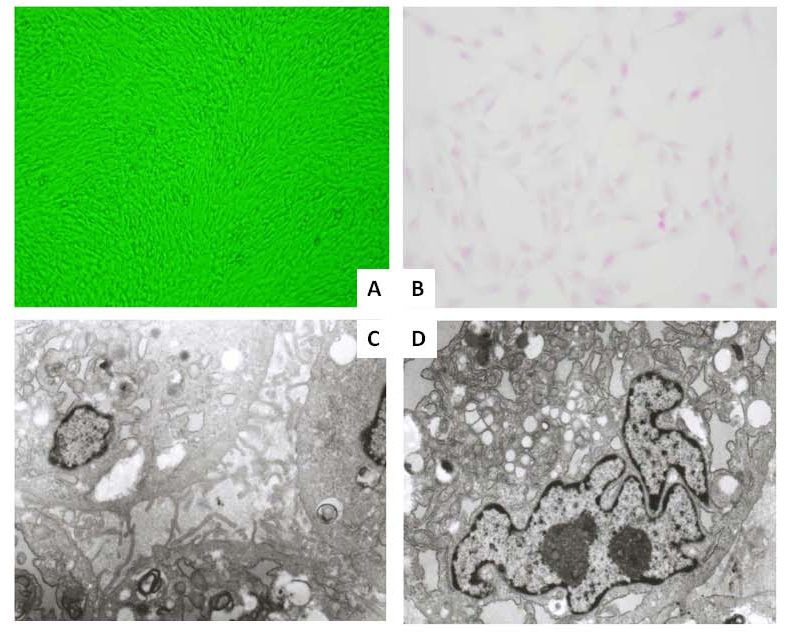
**Cloned cells of testicular yolk sac tumors adhered and grew in the bundle toward the certain direction arranged with multiple overlapping growths (A, × 100), obvious heteromorphism and different shapes in size with H&E staining (B, × 200)**. Microvilli were noted on the cell surface between cells (C, × 8000), with the irregular nuclear (D, × 10000).

Cloned cells of TYST were passed up to 25 generations with the stable growth and primary characteristics, and adhered on the bottom of culture bottle in shapes of short spindle or polygon. Cells lost cell contact inhibition and grew in the bundle toward the certain direction arranged with multiple overlapping growths (Figure 3A). Cells showed obvious heteromorphism and different shapes in size (Figure 3B). Microvilli were noted on the cell surface and the number of rough surfaced endoplasmic reticulum and free ribosome increased, while the number of mitochondria reduced. The nuclear membrane in irregular shapes was edged by decreased heterochromatin (Figure 3c and 3d).

**Figure 4:**
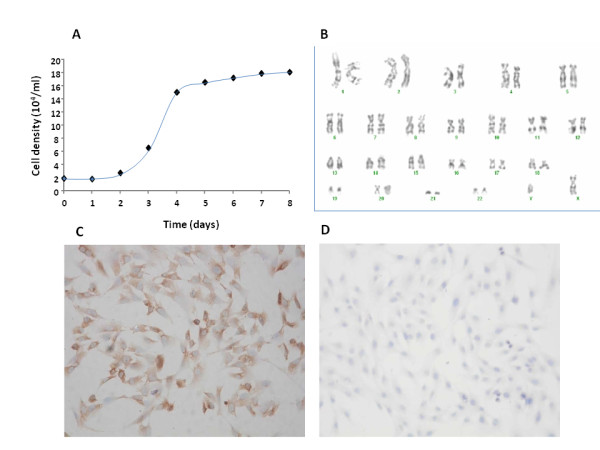
**Cloned testicular yolk sac tumor cell growth curve during 8 day culture (A), with some abnormal chromosomes (B, X400), or positive expression of alpha- fetoprotein (C, × 200), rather than beta-subunit human chorionic gonadotrophin (D, × 200)**.

Cloned TYST cell growth curve demonstrated that there was a rapid growth of cells 2-4 days after the culture, followed by a small and consistent growth from 5 days and on (Figure 4A). Proliferation cycle time of cloned cells was about 30 hours, during which cloned cells were doubled. The number of cloned cell chromosomes fluctuated from 39 to 97, and the modal number was 46. There was no isochromosome of 12p, i(12p) in G-banding, and some chromosomes in cloned cells had abnormal structures similar to those in the primary TYST cells (Figure 4B). Cloned cells had positive expression of AFP (Figure 4C), rather than β-hCG (Figure 4D). DNA index of cloned cells was 1.3, as compared with the normal range between 0.9 with 1.1. DNA ploidy analysis showed aneuploidy, and DNA cycle analysis showed that the G0-G1 accounted for 80% (normal value 80%), G2-M for 17.5% (normal value 10%), and S phase for 2.5% (normal value 10%). The purification rate of cloned TYS cells reached about 60% in the 20th generation cells in double agar for 14 days.

**Figure 5:**
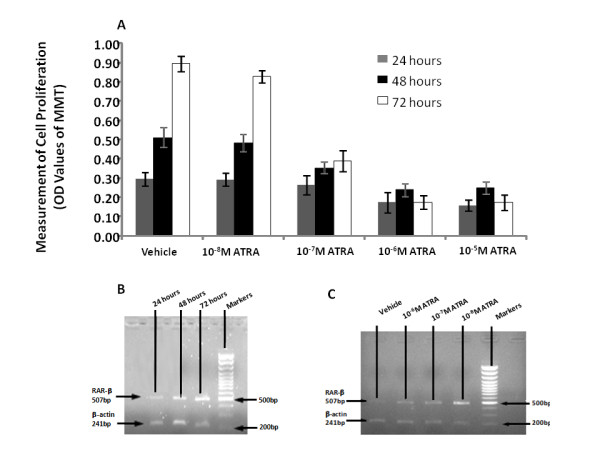
**Inhibitory effects of all-trans-retinoic acid (ATRA) at different concentrations on cell proliferation (A) measured by values of the optimal density (OD) for 24, 48 and 72 hours, or the expression of RAR-β mRNA during 24-72 hours after the treatment with ATPA at concentration of 10^-6 ^M (B) and treated with different concentrations of ATRA at 48 hours (c)**.

ATRA in the concentrations of 10^-7 ^M had significant inhibitory effects on cell proliferation at 48 and 72 hours (inhibitory rates: 31 and 56%, respectively, p < 0.05 and 0.01), 10^-6 ^M at 24, 48 and 72 hours (inhibitory rates: 41, 53 and 80%, p < 0.01, respectively), or 10^-5 ^M at 24, 48 and 72 hours (inhibitory rates: 46, 51 and 81%, p < 0.01, respectively), as shown in Figure 5A. The expression of RAR-β mRNA significantly increased from 48 hours and on as well as by time after the treatment with ATPA at concentration of 10^-6 ^M (Figure 5B) and with the increased concentrations of ATRA at 48 hours (Figure 5C).

**Figure 6:**
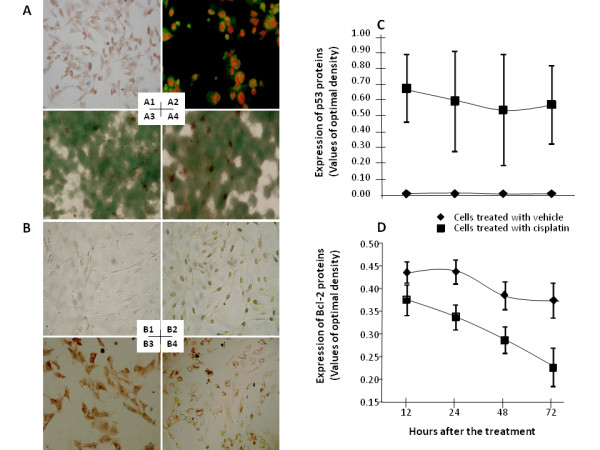
**All closed cells used for the measurement of cisplatin effects were alpha- fetoprotein -stained positive (A1)**. Apoptotic cells were detected with AO/EB staining (A2). Increased TUNEL-positive cells were detected 12 hours after the treatment with cisplatin (A4) or vehicle (A3). The expression of p53 and Bcl-2 proteins was altered 12 hours and on after the treatment with vehicle (B1 and B3) or cisplantin (B2 and B4), respectively. Values of optimal density of p53 (C) and Bcl-2 protein staining (D) were calculated during 12 and 72 hours after cisplatin treatment.

Closed cells used for the measurement of cisplatin effects were AFP-stained positive (Figure 6-A1). Karyopyknosis, nuclei gradually shrank, or apoptotic bodies of apoptotic cells was shown in Figure 6-A2. As compared with those with vehicle (Figure 6-A3), the number of TUNEL-positive cells increased 12 hours after the treatment with cisplatin (Figure 6-A4). Apoptotic index significantly increased 12 (14.7 ± 3.4), 24 (20.2 ± 1.6), 48 (26.9 ± 3.4) and 72 hours (34.2 ± 3.6) after the co-culture with cisplatin, as compared with that with vehicle (6.1 ± 1.8, p < 0.05 or 0.01, respectively). The expression of p53 proteins increased from 12 hours and on after the treatment with cisplantin (Figure 6-B2), while the expression of Bcl-2 proteins decreased and re-distributed from 48 hours (Figure 6-B4), as compared with those with vehicle (Figure 6-B1 and 6-B3). Significance differences of optimal density of p53 and Bcl-2 protein staining were noticed 12 and 24 hours after cisplatin treatment, as shown in Figure 6C and 6D, respectively. There were significantly positive and negative correlations between p53 and apoptotic index (r = 0.387, P = 0.006) and between Bcl-2 and apoptotic index (r = -0.888, P = 0.000), respectively.

## Discussion

Testicular germ cell tumors account for over 95% of all testicular neoplasms and the incidence was doubled worldwide over past decades up to 7.5/100,000 [13], while pediatric germ cell tumors account for 60-75% of pediatric testicular tumors [14], mostly as yolk sac tumor. The in vivo tumor model by the subcutaneous transplantation of tumor cells is accepted due to convenient operation and easy observation [15]. The current study established the TYST model by the passage from the primary TYST cells to the 7th generation in total 95 nude mice during 15 months. The tumor formation rate increased by the development of generations and the average period of tumor formation was about 30 days, similar to pathological characteristics of human TYST [16]. Even though, it is also a challenging to establish the animal model to match the exact schedule and nature of human tumor, which needs more and further investigations. TYST cells were developed into the phase between poorly differentiated and undifferentiated states, with the embryonic or primordial activity. We noted the local differentiation and growth of human TYST cells from the columnar to glandular-like or goblet cells-like cells, while few metastasis in the TYST model. A number of animal models related to TYST have been investigated and vary among the types and mutations of tumor and its cells, e.g. animal model of human disease based on yolk sac carcinoma (endodermal sinus tumor) (49).

AFP was considered as a mark for the presence of YST, which was initially found in the fetal yolk sac [17], embryonic cells [18] and the gastrointestinal tract [19]. There was a clear correlation between AFP and malignant germ cell tumor [20-23]. AFP was used as a monitoring marker of closed TYST cells and formed in vivo tumor in the resent study. Data from the present study demonstrated that AFP proteins was expressed in isolated primary TYST cells, closed TYST cells, pre-transplanted cells, and formed tumor in animals. In addition, we also noted that TYST cells were positive to PLAP, which was significantly over-expressed on the surface of various solid tumor cells [24] and CK as a unique characteristic of tumor cells [25]. PLAP was also considered as a drug design target for non-invasive cancer imaging and therapy [26]. It indicates that the catalysis of the hydrolysis and transphosphorylation of phosphate monoesters is involved in the main metabolism of TYST cells. PLAP, as a classic marker for germ cell tumors through combination of placental membrane glycoprotein with oligosaccharides phosphatidylinositol [24,27], was over-expressed in seminoma, embryonal carcinoma and YST with about 90% positive rate [28]. We also found that chromosomes for tumor identification in each passage met nature of the primary tumor, as previously reported [29].

We initially cloned and established the TYST cell line confirmed by the histology, ultra-structure, growth kinetics and expression of specific proteins and found biological characteristics of cloned cells were similar to the yolk sac tumor. Cloned cells also represented clear microvilli on cell surfaces up to 25 generations, and possessed strong proliferation. The characteristic cytogenetic abnormality in adult testicular germ cell tumors is the invariable gain of material from chromosome 12p [30], so called isochromosome 12p, i(12p), measured by both conventional cytogenetic and molecular cytogenetic techniques, while the abnormal chromosome of i(12p) hardly occurred in infants [31,32]. Our results on i(12p) from the analysis of G-banding were similar to the findings from previous studies [33].

ATRA as natural and synthetic derivatives of vitamin A can bind to the nuclear receptors, involved in the regulation of embryonic development, organogenesis, cell homeostasis, cell growth, differentiation and apoptosis [34-37]. ATRA was used to treat and prevent various types of human cancers and the sensitivity of cancer cells to ATRA was considered as an indicator of the development of ATRA resistance [38]. The cellular sensitivity to ATRA was suggested to be increased upon inactivation of SAG E3 ubiquitin ligase via genetic deletion or pharmacological inhibition [36]. We found that the concentration of ATRA at 10^-5 ^M had inhibitory effects of about 50% on TYST cell proliferation at 24 hours and 10^-7 ^M at 72 hours. It seems that TYST cells were more sensitive to ATRA which had significantly inhibitory effects on cell proliferation in the concentrations of 10^-7 ^M at 48 hours and 10^-6 ^M at 24 hours, as compared with other cells, e.g. peripheral blood mononuclear cells and CD4+ T cells (>10^-4 ^M [39]), human A375 melanoma cells (>10^-5 ^M [40]), breast cancer cells (> > 10^-5 ^M [41]), or small intestinal epithelium (>10^-5 ^M [42]). The further study is needed to confirm the variation of drug sensitivity between different tumor cells. The binding of ATRA with the receptor leads to alterations of gene expressions, followed by a transport to the nucleus and an activation of ATRA-associated signal transduction [43]. We believe over-expression of ATRA receptors on TYST cells may be responsible for the high sensitivity of TYST cells to ATRA, while the binding of ATRA per se could down-regulate the expression of the receptor.

Cisplatin-based chemotherapy was recently proposed to be an alternative of therapies for patients with germ cell tumors, due to the early vascular toxicity of chemotherapy in the endothelium and production of von Willebrand factors [44]. It was also found that cisplatin might have the direct effects on tumor cell to induce DNA damage and activation of JNK/SAPK pathway [45]. Data from the present study demonstrated that cisplatin induced apoptosis of TYST cells through the activation of p53 expression and down-regulation of Bcl- expression. Cisplatin could change the configuration of P53, induce DNA damage, and inhibit cell proliferation by blocking the cell cycle in G1 phase to apoptosis [46]. Cisplatin might change the distribution Bcl around the nucleus or reduce the connection, transport, or formation of nuclear pore complex, and the maintenance of the nuclear envelope, probably associated with the development of cisplatin resistance in TYST cells [47,48].

In conclusion, we cloned human TYST cells and investigated pathological characteristics of cloned cells in the in vitro and in vivo conditions, confirmed by the histology, ultra-structure, growth kinetics and expression of specific proteins and found biological characteristics of cloned cells were similar to the yolk sac tumor. Human TYST cells could be differentiated from the columnar to glandular-like or goblet cells-like cells. Chromosomes for tumor identification in each passage met nature of the primary tumor. TYST cells were more sensitive to ATRA. Cisplatin induced apoptosis of TYST cells through the activation of p53 expression and down-regulation of Bcl- expression. Thus, we believe that cloned TYST cells and the animal model developed are useful to understand the molecular mechanism of TYST cells and screen new drug candidates for the disease.

## Competing interests

The authors declare that they have no competing interests.

## Authors' contributions

JFZ, CDC, HCZ, JHS, HZ, XKL, LQ, XZB, JL and WQL performed and participated in analysis of laboratory experiments data. XMC, XDW, JFZ, CDC, HCZ, JHS participated in the design of experiments. XMC provided administrative support and funded experiments. JFZ, CDC, HCZ, XDW and XMC drafted the manuscript. All authors have read and approved the final manuscript.
